# 4D Multiphase steady state imaging with contrast (MUSIC) enhancement using ferumoxytol: a new paradigm in pediatric congenital heart disease

**DOI:** 10.1186/1532-429X-18-S1-O113

**Published:** 2016-01-27

**Authors:** Kim-Lien Nguyen, Fei Han, Daniel Z Brunengraber, Stanislas Rapacchi, Ihab Ayad, Gary M Satou, Peng Hu, J Paul Finn

**Affiliations:** 1 grid.19006.3e0000000096326718Department of Radiological Sciences, David Geffen School of Medicine at UCLA, Los Angeles, CA USA; 2 Division of Cardiology, David Geffen School of Medicine at UCLA and VA Greater Los Angeles Healthcare System, Los Angeles, CA USA; 3 grid.19006.3e0000000096326718Department of Anesthesiology, David Geffen School of Medicine at UCLA, Los Angeles, CA USA; 4 grid.19006.3e0000000096326718Division of Pediatric Cardiology, David Geffen School of Medicine at UCLA, Los Angeles, CA USA

## Background

CMR in children with congenital heart disease (CHD) often requires expert physician supervision and long examination times. Further, the complex anatomy may not be fully defined with limited 2D slices at the time of image acquisition and missing slice orientations cannot be reconstructed retrospectively. 4D MUSIC (1) generates isotropic high-resolution 3D images over multiple, independent cardiac phases without breath holding. We aim to evaluate this technique in patients with complex CHD and compare it to breathe held contrast enhanced MRA (CE-MRA) and 2D cine.

## Methods

Forty-three children (age 3 days to 19 years; weight 1.2 kg to 62 kg) with suspected or known complex CHD underwent ferumoxytol-enhanced CMR on a 3.0T MRI system between 2013 and 2015. Two readers with advanced CMR training scored the diagnostic image quality of named intra-cardiac structures and multiple vascular segments including the coronary arteries using a four-point scale. Surgery, correlative imaging or autopsy confirmed MUSIC MRI findings. Quantitative volumetric measurements derived from 4D MUSIC and conventional 2D cine images were compared.

## Results

There were no adverse reactions to ferumoxytol. Intra-cardiac and vascular anatomy were significantly better visualized (p < 0.001) with MUSIC than with breath held CE-MRA. Coronary artery anatomy was routinely visualized with high confidence on MUSIC images. Quantitative volumetric measurements derived from 4D MUSIC and conventional 2D cine imaging were strongly correlated (r = 0.99, p < 0.001) and concordance with correlative imaging, surgical findings and /or autopsy results was excellent.

## Conclusions

Ferumoxytol-enhanced 4D MUSIC is superior to breath-held CEMRA in children with CHD and may serve as a comprehensive technique for high resolution anatomic and dynamic imaging. The implications for safe and rapid streamlining of data acquisition in pediatric CHD, and for diagnostic evaluation of patients too unstable for breath holding are significant.

**Figure 1:**
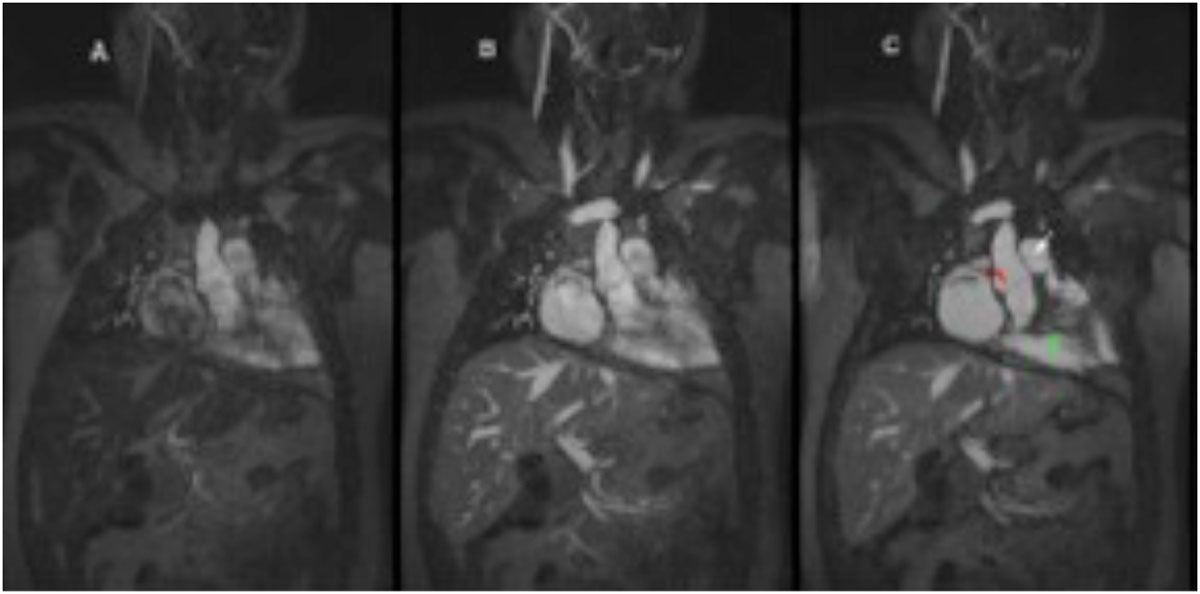
**(a, b, c). Breath held first pass (A), breath held steady state (B) and MUSIC (C) images in an 18 y.o. female with repaired Tetralogy of Fallot**. The arrows point to well defined aortic (red) and pulmonary (white) valve leaflets as well as thickened right ventricular trabeculae (green).

